# Brain-Derived Neurotrophic Factor Reduces Long-Term Mortality in Patients With Coronary Artery Disease and Chronic Kidney Disease

**DOI:** 10.3389/fcvm.2022.881441

**Published:** 2022-06-21

**Authors:** Cheng-Yueh Hsu, Wayne Huey-Herng Sheu, I-Te Lee

**Affiliations:** ^1^Master of Public Health, Johns Hopkins Bloomberg School of Public Health, Johns Hopkins University, Baltimore, MD, United States; ^2^Department of Medical Education, Linkou Chang Gung Memorial Hospital, Taoyuan, Taiwan; ^3^Department of Internal Medicine, Taipei Veterans General Hospital, Taipei, Taiwan; ^4^School of Medicine, National Yang Ming Chiao Tung University, Taipei, Taiwan; ^5^Division of Endocrinology and Metabolism, Department of Internal Medicine, Taichung Veterans General Hospital, Taichung, Taiwan; ^6^School of Medicine, Chung Shan Medical University, Taichung, Taiwan

**Keywords:** brain-derived neurotrophic factor, chronic kidney disease, cohort, coronary artery disease, interaction, mortality

## Abstract

**Objectives:**

Chronic kidney disease (CKD) is a risk factor for coronary artery disease (CAD). We examined the effects of circulating brain-derived neurotrophic factor (BDNF) on long-term mortality in patients with CAD and CKD.

**Materials and Methods:**

We enrolled patients with established CAD in the present study. Serum BDNF and estimated glomerular filtration rate (eGFR) were assessed after overnight fasting. All-cause mortality served as the primary endpoint.

**Results:**

All 348 enrolled patients were divided into four groups according to their median BDNF level and CKD status, defined according to eGFR <60 mL/min/1.73 m^2^. Forty-five patients reached the primary endpoint during the median follow-up time of 6.0 years. Kaplan-Meier survival analysis indicated that the group with low BDNF and CKD had a significantly higher mortality rate than the other three groups (log-rank test *p* < 0.001). Compared to the high BDNF without CKD group, the low BDNF with CKD group had a hazard ratio (HR) of 3.186 [95% confidence interval (CI): 1.482–6.846] for all-cause mortality according to the multivariable Cox proportional hazard regression analysis after adjusting for age and urine albumin-creatinine ratio (*p* = 0.003). Furthermore, there was a significantly interactive effect between BDNF and CKD status on the risk of the primary endpoint (odds ratio = 6.413, 95% CI: 1.497–27.47 in the multivariable logistic regression model and HR = 3.640, 95% CI: 1.006–13.173 in the Cox regression model).

**Conclusion:**

We observed a synergistic effect between low serum BDNF levels and CKD on the prediction of all-cause mortality in patients with CAD.

## Introduction

Coronary artery disease (CAD) is a leading global cause of mortality not only in the general population but also in patients with chronic kidney disease (CKD) (1–4). CKD has resulted in a heavy health burden in Taiwan, where a high prevalence and incidence of CKD have long been reported (5, 6). CKD is associated with arterial calcification and stiffness caused by uremic effects and electrolyte imbalance (2, 7). CKD is also accompanied by hypertension, dyslipidemia, oxidative stress, and chronic inflammation, all of which may accelerate the progression of atherosclerosis (8, 9). However, traditional risk factors cannot fully explain the shared pathogenesis between CAD and CKD (10). Therefore, the identification of new circulating biomarkers is warranted to evaluate and predict mortality in patients with CAD and CKD.

Brain-derived neurotrophic factor (BDNF) is a neurotrophic factor that protects the growth of neurons and synaptic plasticity (11–14). In addition to its neuroprotective effects (15, 16), BDNF is also important for cardiac development (17–20). A reduction in circulating BDNF levels is associated with chronic inflammation and oxidative stress (21–24). Tropomyosin-related kinase receptor B (TrkB), a receptor of BDNF, is expressed on endothelial cells and vascular smooth muscles (17, 25), and BDNF has been reported to be associated with a reduction in pulse pressure (26, 27). BDNF may be protective against mortality, most likely via TrkB signaling (28–30). An animal study conducted in rodents revealed that BDNF could prevent damage to glomerular podocytes via TrkB signaling (31), which is also thought to be essential for early renal cell differentiation, kidney structure formation, and renal function maintenance (32, 33). A low circulating BDNF concentration was reported to significantly predict the incidence of CKD in the Hyogo Sleep Cardio-Autonomic Atherosclerosis Study (34).

Although BDNF has been reported to be protective against adverse cardiovascular events and mortality in longitudinal studies (29, 35), the composite role of BDNF and CKD in the prediction of all-cause mortality in patients with established CAD remains unclear. Since BDNF has benefits in regard to the common risk factors for CAD and CKD, including arterial stiffness (26, 27), chronic inflammation (22, 23), and oxidative stress (21, 24), we hypothesized that BDNF acts as a mediator in the relationship between the heart and kidney. Therefore, in this study, we examined the composite effects of BDNF and CKD on all-cause mortality in patients with established CAD.

## Materials and Methods

### Study Participants

In this observational cohort study, we prospectively and continuously screened candidates with angina who were hospitalized for selective angiography at Taichung Veterans General Hospital since April 2009. To effectively investigate the associated risk factors, patients with the following conditions were excluded from the present study: (a) history of known diabetes mellitus (DM), (b) history of any cancer, (c) history of any autoimmune disease, (d) history of any psychiatric disease, (e) currently active infection, (f) symptomatic congestive heart failure ≥ class 3 based on the criteria from the New York Heart Association (36), and (g) drug addiction or alcoholism. Eligible patients with established CAD according to clinical diagnosis and coronary angiography findings between April 2009 and December 2016 were enrolled in this study. Patients who required surgery for coronary artery bypass graft were excluded. An outpatient appointment was scheduled for a baseline assessment after percutaneous coronary intervention and medical treatment. Finally, a total of 348 adult patients completed the baseline assessment.

### Procedures

Body height and body weight were measured after the patients had removed their shoes and any heavy clothing. Blood pressure was measured using the Carescape V100 DINAMAP^®^ Vital Signs Monitor (GE Healthcare, Milwaukee, WI, United States) after the patients rested in a sitting position for 10 min. Morning urine samples were collected for the detection of albumin and creatinine. Fasting blood samples were collected after anthropometric measurements to detect BDNF, glucose, hemoglobin A1c (HbA1c), creatinine, and lipid profiles, including total cholesterol, low-density lipoprotein (LDL) cholesterol, high-density lipoprotein (HDL) cholesterol, and triglycerides. After the assessment of the baseline characteristics, the occurrence of the primary endpoint, all-cause mortality, was followed up through August 31, 2019. Death registration information was obtained from the Ministry of Health and Welfare, Executive Yuan, Taiwan.

### Laboratory Assessments

Serum samples were prepared by placing the blood in a serum separator tube for approximately 30 min at room temperature followed by centrifugation. Serum samples were stored at −80°C and were first thawed for this study. Human mature BDNF in the serum was measured using an immunoassay kit (DBD00, R&D Systems, Minneapolis, MN, United States); the precision of the BDNF measurement was as follows: intra-assay coefficient of variation (CV) of 6.2% and inter-assay CV of 8.1%. Serum lipid profiles and creatinine levels were measured using the commercially available kits (Beckman Coulter, Fullerton, CA, United States). The estimated glomerular filtration rate (eGFR) was calculated using the following formula: 186 × (serum creatinine [mg/dL])^–1.154^ × (age [years]) ^–0.203^ (×0.742, for women), according to the Modification of Diet in Renal Diseases Equation (37). The urine albumin-creatinine ratio (UACR) was defined as the ratio of urine albumin (mg) to urine creatinine (g). Baseline CAD was defined as the presence of one or more of the following conditions: (a) history of myocardial infarction (MI), (b) history of coronary revascularization, or (c) a coronary lesion with lumen narrowing ≥50% according to angiography. Hypertension was defined as the presence of any the following condition: (a) history of antihypertensive agent use, (b) systolic blood pressure ≥140 mmHg, or (c) diastolic blood pressure ≥90 mmHg on the day of visit. Plasma glucose levels were measured by the oxidative peroxidase method (Wako Diagnostics, Tokyo, Japan). HbA1c levels were measured using boronate affinity high-performance liquid chromatography (NGSP certified, Primus Corp., Kansas City, MO, United States).

### Statistical Analysis

Continuous variables are reported as mean ± standard deviation, and categorical variables are reported as numbers with percentages. Pearson’s correlation coefficient analysis was used to determine the relationship between serum BDNF levels and eGFR. To examine the synergistic effect of BDNF and CKD status on all-cause mortality risk in patients with CAD, we divided all the enrolled patients into four groups according to serum BDNF level and CKD status, as follows: high BDNF without CKD, low BDNF without CKD, high BDNF with CKD, and low BDNF with CKD. The cutoff point to define low BDNF and high BDNF was the median value of 24.58 ng/mL. CKD was defined as an eGFR <60 mL/min/1.73 m^2^ (38).

To ascertain significant differences among the four groups, we used the Kruskal–Wallis test to examine the continuous variables, considering the relatively small sample size of the four groups. Chi-squared tests were used to examine the categorical variables. To assess the association between baseline risk factors and mortality, we compared the continuous variables by Mann–Whitney test and the categorical variables by chi-squared test between the mortality and survival groups in a univariable analysis.

The risk of reaching the primary endpoint was examined using Kaplan-Meier survival analysis, and statistically significant differences among the groups were detected using the log-rank test. Multivariable Cox proportional hazards regression analyses were performed to evaluate the risk of the primary endpoint according to the groups categorized by serum BDNF level and CKD status. The covariates in the Cox regression analyses included age and sex for the model 1 and the significant predictors for mortality detected in the univariable analysis for model 2. Multivariable logistic regression and Cox regression analyses were conducted to evaluate the interactive effect between BDNF and CKD on mortality after adjusting for the significant predictors of mortality detected in the univariable analysis. A two-sided *p-*value < 0.05 was considered to be statistically significant. Statistical analysis was conducted using SPSS v22.0 (IBM, Armonk, NY, United States).

## Results

**FIGURE 1:**
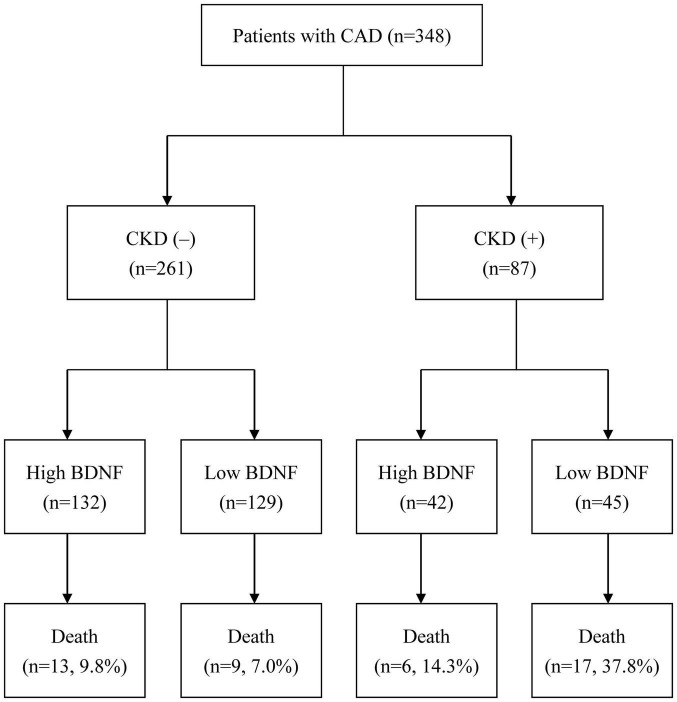
Flow diagram of the enrollment and analysis of the study participants (CAD, coronary artery disease; CKD, chronic kidney disease; BDNF, brain-derived neurotrophic factor).

**FIGURE 2:**
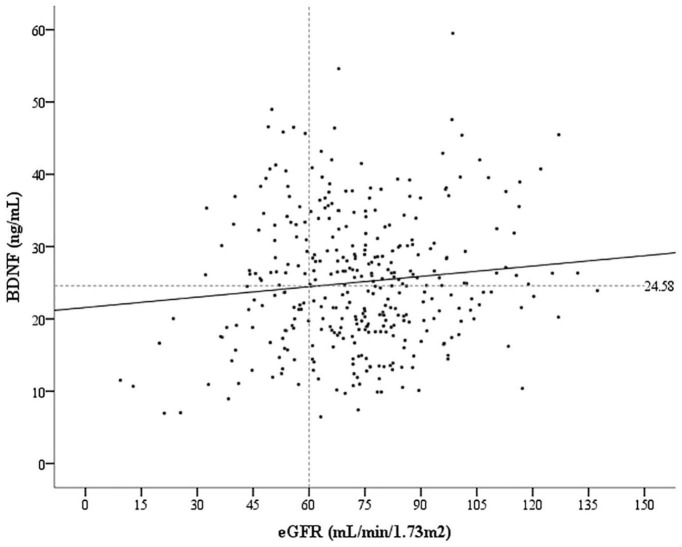
Correlation between the serum brain-derived neurotrophic factor (BDNF) levels and estimated glomerular filtration rate (eGFR). Pearson correlation coefficient = –0.108, *p* = 0.045.

A total of 348 patients with CAD were enrolled in the present study. There was a positive correlation between serum BDNF levels and eGFR (correlation coefficient = 0.108, *p* = 0.045; Figure 1). A total of 45 primary endpoint events occurred during the median follow-up time of 6.0 years. Based on their serum BDNF levels and CKD status, all the patients were divided into four groups (Figure 2): 132 patients were in the high BDNF without CKD group (13 primary endpoints, 9.8%), 129 patients were in the low BDNF without CKD group (9 primary endpoints, 7.0%), 42 patients were in the high BDNF with CKD group (6 primary endpoints, 14.3%), and 45 patients were in the low BDNF with CKD group (17 primary endpoints, 37.8%). The baseline characteristics of these four groups are presented in Table 1. There were significant differences among these four groups according to age, UACR, eGFR, and BDNF (all *p-*values < 0.001), while none of the other assessed characteristics reached statistical significance, as determined by the Kruskal–Wallis test.

**TABLE 1:** The baseline characteristics of the enrolled patients categorized by eGFR of 60 mL/min/1.73 m^2^ and median serum BDNF (24.58 ng/mL).

	High BDNF without CKD (*n* = 132)	Low BDNF without CKD (*n* = 129)	High BDNF with CKD (*n* = 42)	Low BDNF with CKD (*n* = 45)	*p-*value
Age (years)	59.6 ± 11.5	60.1 ± 10.6	64.3 ± 10.6	72.6 ± 9.1	<0.001
Male, *n* (%)	119 (90.2%)	115 (89.1%)	35 (83.3%)	41 (91.1%)	0.621
Current smoker, *n* (%)	23 (17.6%)	15 (11.6%)	6 (14.6%)	2 (4.4%)	0.139
BMI (kg/m^2^)	26.7 ± 4.0	25.8 ± 3.4	26.5 ± 3.9	25.1 ± 2.9	0.125
Systolic BP (mmHg)	126.9 ± 18.1	125.6 ± 17.1	132.2 ± 20.3	132.6 ± 18.2	0.096
Diastolic BP (mmHg)	75.5 ± 10.0	73.3 ± 10.6	74.9 ± 10.5	72.2 ± 9.2	0.302
Fasting glucose (mmol/L)	5.4 ± 0.7	5.3 ± 0.6	5.3 ± 0.7	5.4 ± 1.2	0.328
HbA1c (%)	5.9 ± 0.6	5.9 ± 0.6	6.0 ± 0.6	6.1 ± 0.6	0.564
Total cholesterol (mmol/L)	4.3 ± 1.1	4.2 ± 1.0	4.1 ± 0.9	4.4 ± 1.0	0.535
HDL cholesterol (mmol/L)	1.2 ± 0.3	1.2 ± 0.3	1.2 ± 0.3	1.2 ± 0.3	0.709
LDL cholesterol (mmol/L)	2.5 ± 0.9	2.4 ± 0.8	2.2 ± 0.9	2.6 ± 0.9	0.424
Triglyceride (mmol/L)	1.5 ± 0.8	1.3 ± 0.6	1.8 ± 1.4	1.5 ± 0.6	0.153
BDNF (ng/mL)	32.0 ± 6.5	18.1 ± 4.2	33.5 ± 6.8	17.0 ± 4.7	<0.001
eGFR (mL/min/1.73 m^2^)	82.0 ± 16.8	81.4 ± 14.4	50.4 ± 6.9	45.3 ± 12.8	<0.001
UACR ≥30 mg/g, *n* (%)	14 (10.6%)	27 (20.9%)	18 (42.9%)	11 (24.4%)	<0.001
Hypertension, *n* (%)	126 (95.5%)	127 (98.4%)	40 (95.2%)	42 (93.3%)	0.369
Antihypertensive agents, *n* (%)	123 (93.2%)	122 (94.6%)	38 (90.5%)	40 (88.9%)	0.570
ACE inhibitors or ARBs, *n* (%)	84 (63.6%)	91 (70.5%)	29 (69.0%)	29 (64.4%)	0.655
Antiplatelet drugs, *n* (%)	131 (99.2%)	125 (96.9%)	41 (97.6%)	42 (93.3%)	0.182
Statins	97 (73.5%)	102 (79.1%)	29 (69.0%)	32 (71.1%)	0.489

*Continuous data are expressed as the mean ± SD and were examined using Kruskal–Wallis test.*

*Categorical data are expressed as the number with percentage and were examined using Chi-Square test.*

*ACE, angiotensin-converting enzyme; ARBs, angiotensin II receptor antagonists; BDNF, brain-derived neurotrophic factor; CKD, chronic kidney disease; BMI, body mass index; BP, blood pressure; HbA1c, hemoglobin A1c; HDL, high-density lipoprotein; LDL, low-density lipoprotein; eGFR, estimated glomerular filtration rate; SD, standard deviation; UACR, Urine albumin-creatinine ratio.*

Table 2 presents the results of the univariable analysis of the association between risk factors and all-cause mortality. The risk of mortality was significantly different among the four groups categorized by serum BDNF levels and CKD status (*p* < 0.001). The patients in the low BDNF with CKD group had a higher mortality risk (37.8%) than those in the high BDNF without CKD group (9.8%), the low BDNF without CKD group (7.0%), and the high BDNF with CKD group (14.3%). In addition, the proportion of patients aged ≥60 years was higher in the mortality group than in the survival group (80.0 vs. 54.1%, *p* = 0.002). The prevalence of UACR ≥30 mg/g is also significantly higher in the mortality group than in the survival group (35.6 vs. 17.8%, *p* = 0.010). None of the other assessed risk factors exhibited statistically significant differences between the mortality and the survival groups.

**TABLE 2:** Univariable analysis of the association between risk factors and mortality.

	Mortality (*n* = 45)	Survival (*n* = 303)	*p*
Groups categorized by BDNF and CKD			<0.001
High BDNF without CKD (*n* = 132)	13 (9.8%)*	119 (90.2%)*	
Low BDNF without CKD (*n* = 129)	9 (7.0%)*	120 (93.0%)*	
High BDNF with CKD (*n* = 42)	6 (14.3%)*	36 (85.7%)*	
Low BDNF with CKD (*n* = 45)	17 (37.8%)*	28 (62.2%)*	
Age (≥60 years)	36 (80.0%)	164 (54.1%)	0.002
Male	42 (93.3%)	268 (88.4%)	0.446
BMI (≥27 kg/m^2^)	17 (37.8%)	108 (35.6%)	0.911
Current smoker	6 (13.3%)	40 (13.3%)	0.999
Systolic BP (≥130 mmHg)	20 (44.4%)	133 (43.9%)	0.999
Diastolic BP (≥85 mmHg)	3 (6.7%)	44 (14.5%)	0.228
Fasting glucose (≥100 mg/dL)	12 (26.7%)	88 (29.0%)	0.879
HbA1c (≥5.7%)	34 (75.6%)	201 (66.3%)	0.288
Total cholesterol (≥160 mg/dL)	24 (53.3%)	139 (45.9%)	0.438
Low HDL cholesterol^†^	15 (33.3%)	102 (33.7%)	1.000
LDL cholesterol (≥100 mg/dL)	19 (42.2%)	103 (34.0%)	0.362
Triglyceride (≥150 mg/dL)	12 (26.7%)	92 (30.4%)	0.741
UACR (≥30 mg/g)	16 (35.6%)	54 (17.8%)	0.010
Hypertension*^f^*	42 (93.3%)	293 (96.7%)	0.229
Antihypertensive agents*^f^*	41 (91.1%)	282 (93.1%)	0.547
ACE inhibitors or ARBs	32 (71.1%)	201 (66.3%)	0.642
Antiplatelet drugs*^f^*	45 (100.0%)	294 (97.0%)	0.611
Statins	29 (64.4%)	231 (76.2%)	0.130

*Data are expressed as the number with percentage.*

*Indicates the percentage of mortality in each of the four subgroups.

*^†^Indicates HDL cholesterol <50 mg/dL (1.29 mmol/L) in women or <40 mg/dL (1.03 mmol/L) in men.*

*^f^Fisher’s exact test.*

*ACE, angiotensin-converting enzyme; ARBs, angiotensin II receptor antagonists; BDNF, brain-derived neurotrophic factor; CKD, chronic kidney disease; BMI, body mass index; BP, blood pressure; HbA1c, hemoglobin A1c; HDL, high-density lipoprotein; LDL, low-density lipoprotein; eGFR, estimated glomerular filtration rate; SD, standard deviation; UACR, Urine albumin-creatinine ratio.*

**FIGURE 3:**
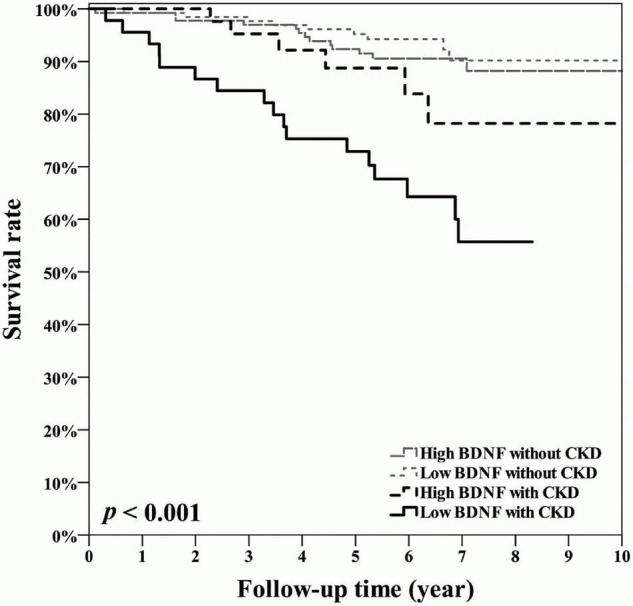
Kaplan-Meier curves showing the survival rates across the four groups, defined based on a median serum BDNF value of 24.58 ng/mL and CKD status defined as an estimated glomerular filtration rate of <60 mL/min/1.73 m^2^. CKD, chronic kidney disease; BDNF, brain-derived neurotrophic factor.

Figure 3 shows that the survival rate was the lowest in the low BDNF with CKD group according to the Kaplan-Meier survival analysis (log rank test: *p* < 0.001). We conducted multivariable Cox regression analyses, as presented in Table 3. The risk of reaching the primary endpoint was significantly higher in the low BDNF with CKD group than in the high BDNF without CKD group (hazard ratio = 3.186, 95% CI: 1.482–6.846, *p* = 0.003) after adjusting for age and UACR which were the significant predictors for mortality in Table 2. We also examined the interaction between BDNF and CKD status on the primary endpoint, and the results are shown in Table 4. The variable BDNF × CKD status was found to contribute to the multivariable logistic regression model (odds ratio = 6.413, 95% CI: 1.497–27.47) and Cox regression model (hazard ratio = 3.640, 95% CI: 1.006–13.173) after adjusting for age and UACR.

**TABLE 3:** Cox proportional hazard regression models for the association between risk factors and mortality.

	Crude			Model 1			Model 2		
	HR	95% CI	*p*	HR	95% CI	*p*	HR	95% CI	*p*
High BDNF without CKD	1.000			1.000			1.000		
Low BDNF without CKD	0.718	(0.307, 1.680)	0.445	0.743	(0.317, 1.740)	0.494	0.681	(0.289, 1.602)	0.378
High BDNF with CKD	1.802	(0.684, 4.747)	0.234	1.693	(0.638, 4.499)	0.291	1.286	(0.473, 3.493)	0.622
Low BDNF with CKD	4.530	(2.199, 9.331)	<0.001	3.374	(1.585, 7.183)	0.002	3.186	(1.482, 6.846)	0.003
Age (≥60 years)				2.270	(1.038, 4.967)	0.040	1.928	(0.882, 4.215)	0.100
Male				2.208	(0.673, 7.243)	0.191			
UACR (≥30 mg/g)							1.973	(1.047, 3.718)	0.036

*BDNF, brain-derived neurotrophic factor; CKD, chronic kidney disease; CI, confidence interval; HR, hazard ratio; UACR, urine albumin-creatinine ratio.*

**TABLE 4:** The interaction between BDNF and CKD status on the primary endpoint*.

	Logistic regression			Cox regression		
	OR	95% CI	*p*	HR	95% CI	*p*
BDNF (high/low)						
High BDNF (reference group)	1.000			1.000		
Low BDNF	0.614	(0.247, 1.523)	0.292	0.681	(0.289, 1.602)	0.378
CKD	0.994	(0.334, 2.965)	0.992	1.286	(0.473, 3.493)	0.622
BDNF (high/low) × CKD	6.413	(1.497, 27.47)	0.012	3.640	(1.006, 13.173)	0.049

*Adjustment for age and urine albumin-creatinine ratio. BDNF, brain-derived neurotrophic factor; CKD, chronic kidney disease; HR, hazard ratio; OR, odds ratio; CI, confidence interval.

## Discussion

Our main finding is that CKD accompanied by a lower serum BDNF concentration predicted a higher risk of all-cause mortality during a median follow-up time of 6.0 years among the 348 patients with CAD. To the best of our knowledge, this is the first study to ascertain the composite effect of low serum BDNF levels and CKD on long-term mortality. In line with our findings, Kaess et al. (35) reported that a lower serum BDNF level is predictive of a greater cardiovascular risk based on the data from the population in Framingham. Zhou et al. (39) reported that polymorphisms in the BDNF gene are associated with ischemic stroke in a genetic study of Chinese Han patients with large-arterial atherosclerosis. Jiang et al. (29) also reported that a lower circulating BDNF level significantly predicted a higher mortality rate in patients with angina pectoris. However, in these above studies, probably due to the nature of the research purposes, neither the eGFR values nor the CKD status of the study participants were characterized. Given that both the prevalence and incidence of CKD are high in Taiwan (5, 6), it might be clinically meaningful and of public health importance to investigate whether the combination of low BDNF and CKD is associated with higher mortality in patients with CAD.

The results of our analyses showed that both age and UACR were significantly different among the four groups and were also significantly associated with mortality. As age and albuminuria are related to CKD, these factors may be confounders in our analyses (39–42); this may explain the higher proportion of patients with UACR ≥30 mg/g in the mortality group than in the survival group. The above observation is in line with a previous study reporting a high UACR associated with CKD (43).

Patients in the low BDNF and CKD group have an approximately three-fold mortality risk than those in a high BDNF without CKD group after adjusting for the other important risk factors. However, the risks of long-term mortality were not significantly between the high BDNF without CKD group and the high BDNF with CKD group or the low BDNF without CKD group. The finding indicates that there may be a synergistic effect of low BDNF and CKD on long-term mortality. This contention is further supported by the multivariable logistic regression models which revealed that the significant interaction variable BDNF × CKD contributes to the regression model after the other associated risk factors are adjusted.

While several studies have ascertained the protective effect of BDNF on cardiovascular disease and mortality (29, 35, 39), relatively few studies have explored the relationship between BDNF and CKD, especially in patients with CAD. As TrkB is expressed in human kidneys (44), the signaling through this molecule probably contributes to the link between BDNF and renal pathophysiology. TrkB signaling has been reported to be essential in early kidney cell differentiation (32). Garcia-Suarez et al. (33) also suggested that TrkB is a key factor involved in renal structure and function because a significant reduction in glomerular areas, an absence of the macula densa, and an increasing number of extraglomerular mesangial cells were observed in TrkB-deficient mice (33).

Brain-derived neurotrophic factor was found to protect the kidneys from apoptosis induced by endoplasmic reticulum stress in a mouse model (45). Li et al. (31) reported that BDNF repairs podocytes by increasing the length and number of podocyte cell processes using an *in vitro* model of focal segmental glomerulosclerosis. The protective benefits were reflected by the *in vivo* improvement of proteinuria and glomerular lesions after BDNF administration in mice with adriamycin nephropathy. In the human kidney, TrkB is exclusively expressed in podocytes (31), and BDNF probably protects podocytes from injury under different conditions. BDNF is also localized in the processes and cell bodies of podocytes in patients with diabetic nephropathy (46). A lower circulating BDNF level significantly predicted the development of CKD in Japanese individuals in the Hyogo Sleep Cardio-Autonomic Atherosclerosis Study (34). Given the results of these previous studies, BDNF may be protective against kidney injury. Our observational findings on long-term survival can be partly explained by the protective effects of BDNF on the renal and cardiovascular systems. However, we were unable to collect laboratory or dialysis data during the follow-up period in the present study. Notably, decreases in both renal function and circulating BDNF concentration synergistically exacerbated mortality risk. This might be a compensatory mechanism between renal function and the effect of BDNF on long-term protection in patients with CAD. Considering that CKD is highly prevalent in patients with CAD and increases mortality (47), our study suggests that serum BDNF levels can be used as a new clinical indicator to categorize the risk of long-term mortality in patients with CAD and CKD.

Brain-derived neurotrophic factor mRNA is expressed in several human tissues, and platelets are a major source of BDNF in the peripheral blood (44, 48, 49). Activated platelets increase the BDNF release, and the short-term administration of antiplatelet drugs can decrease BDNF release from platelets into the serum (49, 50). In contrast, serum BDNF concentrations were not related to stable antiplatelet use in patients with CAD (51). As we enrolled patients with established CAD in this study, most (97.4%) of the participants had taken antiplatelet drugs at baseline assessment. Therefore, the proportion of patients using antiplatelet therapy was not significantly different across the four groups categorized by CKD and serum BDNF levels in this study. Moreover, several limitations of the present study have not been completely addressed. First, we did not directly assess the possible underlying compensatory mechanisms between renal and BDNF functions in patients with CAD. Second, DM might be a confounder in this study because it is associated with nephropathy and mortality. We excluded patients with known DM. Therefore, the results cannot be applied to the population with DM, even though some patients were found to have high glucose levels at baseline in this study. Third, glomerulonephritis associated with immune disease was excluded from the present study. However, renal biopsy was not performed, and the underlying causes of CKD were unknown. Fourth, the use of angiotensin-converting enzyme inhibitors, angiotensin II receptor antagonists, or statins at baseline did not decrease the mortality risk. These non-significant findings might have resulted from changes in medications that were not assessed during the follow-up period. Finally, age and albuminuria are related to CKD, and they might be confounders in our analyses because they were significantly different among the four study groups; these parameters were also significantly associated with mortality. Although we adjusted for these confounders in the multivariable regression analysis, we did not further group the patients according to their age and UACR level because of the limited number of cases. Future larger study cohorts are necessary to ascertain the effects of BDNF and renal function in different populations.

## Conclusion

Chronic kidney disease accompanied by a low serum BDNF concentration is predictive of a significantly high all-cause mortality rate during a median follow-up of 6.0 years among patients with CAD. Further studies are warranted to determine the mechanism and causal relationship between BDNF and CKD on long-term mortality in patients with CAD.

## Data Availability Statement

The datasets used and/or analyzed during the current study are available from the corresponding author upon reasonable request.

## Ethics Statement

The studies involving human participants were reviewed and approved by the Institutional Review Board of Taichung Veterans General Hospital. The patients/participants provided their written informed consent to participate in this study.

## Author Contributions

C-YH, WS, and I-TL contributed to the conception and design of the study. WS and I-TL collected the clinical data and revised the manuscript. C-YH organized the database, performed the statistical analyses, and drafted the manuscript. All authors have contributed to the manuscript and approved the submitted version.

## Conflict of Interest

The authors declare that the research was conducted in the absence of any commercial or financial relationships that could be construed as a potential conflict of interest.

## Publisher’s Note

All claims expressed in this article are solely those of the authors and do not necessarily represent those of their affiliated organizations, or those of the publisher, the editors and the reviewers. Any product that may be evaluated in this article, or claim that may be made by its manufacturer, is not guaranteed or endorsed by the publisher.
